# Frequent microbiological profile changes are seen in subsequent-revision hip and knee arthroplasty for prosthetic joint infection

**DOI:** 10.5194/jbji-8-229-2023

**Published:** 2023-11-03

**Authors:** Robert A. McCulloch, Alex Martin, Bernadette C. Young, Benjamin J. Kendrick, Abtin Alvand, Lee Jeys, Jonathan Stevenson, Antony J. Palmer

**Affiliations:** 1 The Royal National Orthopaedic Hospital, Brockley Hill, Stanmore, Middlesex, HA7 4LP, UK; 2 The Nuffield Orthopaedic Centre, Windmill Road, Headington, Oxford, OX3 7HE, UK; 3 The Royal Orthopaedic Hospital, Bristol Road, Northfield, Birmingham, B31 2AP, UK

## Abstract

A proportion of patients with hip and knee prosthetic joint infection (PJI) undergo multiple revisions with the aim of eradicating infection and improving quality of life. The aim of this study was to describe the microbiology cultured from multiply revised hip and knee replacement procedures to guide antimicrobial therapy at the time of surgery. **Patients and methods**: Consecutive patients were retrospectively identified from databases at two specialist orthopaedic centres in the United Kingdom between 2011 and 2019. Patient were included who had undergone repeat-revision total knee replacement (TKR) or total hip replacement (THR) for infection, following an initial failed revision for infection. **Results**: A total of 106 patients were identified. Of these patients, 74 underwent revision TKR and 32 underwent revision THR. The mean age at first revision was 67 years (SD 10). The Charlson comorbidity index was ≤ 2 for 31 patients, 3–4 for 57 patients, and ≥ 5 for 18 patients. All patients underwent at least two revisions, 73 patients received three, 47 patients received four, 31 patients received five, and 21 patients received at least six. After six revisions, 90 % of patients had different organisms cultured compared with the initial revision, and 53 % of organisms were multidrug resistant. The most frequent organisms at each revision were coagulase-negative *Staphylococcus* (36 %) and *Staphylococcus aureus* (19 %). Fungus was cultured from 3 % of revisions, and 21 % of infections were polymicrobial. **Conclusion**: Patients undergoing multiple revisions for PJI are highly likely to experience a change in organism, with 90 % of patients having a different organism cultured by their sixth revision. It is therefore important to administer empirical antibiotics at each subsequent revision, taking into account known drug resistance from previous cultures. Our results do not support the routine use of empirical antifungals.

obert.mcculloch@nhs.net]Robert A.McCulloch

## 1 Introduction

An increasing proportion of revision hip and knee replacements are performed for prosthetic joint infection (PJI) (ref NJR, https://reports.njrcentre.org.uk/Portals/0/PDFdownloads/NJR 20th Annual Report 2023.pdf, last access: 1 May 2023). PJI gives rise to significant morbidity and expense (Kurtz et al., 2012; Parisi et al., 2017). Reported rates of infection control following revision surgery range from 67 % to 100 % (Gerritsen et al., 2021; Pangaud et al., 2019). Risk factors for persistent infection include patient morbidity, previous revision for PJI (Kheir et al., 2017), and specific organisms such as *Enterococcus* species and *Candida* (Citak et al., 2019; Houdek et al., 2015).

The principles of PJI management are surgical debridement and exchange of implants, with targeted local and systemic antibiotic therapy guided by multidisciplinary team care. Antibiotic regimes may be informed by previous microbiological results; however, it can frequently occur that no organism is cultured (culture-negative) or the infective organism may be different on subsequent presentations. Zmistowski et al. (2013) reported that only 31.5 % of patients cultured the same organisms at repeat sampling in their series.

The change in organism profile has not been well described for patients undergoing sequential revision procedures for hip and knee PJI. An improved understanding of this would guide perioperative antibiotic treatment. The authors hypothesize that the incidence of a changing microbiological profile increases with the number of revision procedures performed. Thus, our aims were to (i) determine the proportion of patients undergoing multiple-revision hip and knee procedures for PJI who experience a change in the organisms cultured and (ii) to explore whether the proportion of polymicrobial infections, multiresistant organisms, and fungal infections increased with subsequent revisions.

## 2 Patients and methods

This was a retrospective study in which consecutive patients were identified from the local databases of two specialist orthopaedic hospitals in the United Kingdom. The project was approved by local committees. Between January 2011 and May 2019, patients were identified who had been admitted for revision hip or knee arthroplasty with an indication of recurrent PJI following an initial debridement and implant retention, single-stage revision, or two-stage revision. Data collection included patient demographics, American Society of Anaesthesiology (ASA) score, Charlson comorbidity index, dates of surgery, description of procedure, microbiology results (including antibiotic sensitivity), and patient outcomes. A systematic sampling method was undertaken in theatre to minimize the risks of contamination. A minimum of five samples were sent for microbiological analysis at each procedure and antibiotics were withheld prior to surgery, unless the patient was systemically septic. All cases and were Gram-stained, cultured by direct and enrichment methods for 15 d along with antibiotic susceptibility testing. Fungal cultures were performed in all cases as routine. Follow-up protocol in both hospitals for patients managed with PJI is clinic review for the first 2 years postoperatively within a specialist multidisciplinary clinic (frequency would be dependent on various factors) and then clinician's preference thereafter.

The definition of PJI was as per the Musculoskeletal Infection Society (MSIS) guidelines (Parvizi et al., 2018). A change in microbiology was defined when a different pathogen was grown on two specimens compared with previous intraoperative cultures. We considered there to have been a likely change in organism of coagulase-negative *Staphylococcus* if there were two or more changes in the antimicrobial sensitivities, consistent with other studies (Young et al., 2023). We have reported these separately from the main results. A negative culture result (when other MSIS criteria for infection were fulfilled) was categorized as an unchanged microbiological profile. Multidrug resistance was defined as lack of susceptibility to at least one agent in three or more classes of antibiotics (Magiorakos et al., 2012). Polymicrobial infection was defined as more than one organism cultured from a single procedure. Descriptive statistics were used to report the data. Continuous data were reported as means and standard deviations (SDs), whereas proportions were reported as absolute values and percentages.

## 3 Results

The cohort consisted of 106 patients who underwent at least two revisions of a prosthetic knee or hip for infection (74 knees and 32 hips). The mean age at first revision was 67 years (SD 10.0); 66 patients were male and 40 were female. The Charlson comorbidity index was 0–2 for 31 (29 %) patients, 3–4 for 57 (53 %) patients, and ≥ 5 for 18 (17 %) patients. The mean time from the primary joint replacement to first revision for infection was 67 months (SD 73). The mean interval between surgeries decreased with each subsequent revision (Table 1). All patients underwent at least two revisions, 73 patients received three, 47 patients received four, 31 patients received five, and 21 patients received at least six.

A change in the organisms cultured took place in 50.7 % of all procedures during the study period (Table 2). Once a patient had undergone six revisions for infection, 90 % (19 of 21) of patients had different organisms cultured compared with the initial revision (Table 3). Two patients who underwent six revisions had only one organism cultured throughout their surgeries, and this was *Staphylococcus epidermidis* in both instances.

The most frequent causative organisms were coagulase-negative *Staphylococcus* (137 of 384; 36 %) and *Staphylococcus aureus* (73 of 384; 19 %) (Table A1 in the Appendix). The proportion of organisms with multidrug resistance increased with each revision, representing more than half of organisms (8 of 15 positive cultures) following six revisions (Table 4). Fungus was cultured from 3 % (11 of 384) of revisions performed in this cohort, and 17 % (66 of 384) of infections were polymicrobial. The incidence of fungal infection and polymicrobial infection did not increase with the number of revisions performed.

Culture of the same organism in subsequent procedures with a change in two or more of the sensitivities on the antibiogram took place in 15 cases. Of these, coagulase-negative *Staphylococcus* had the highest incidence of a change in sensitivity (eight cases) followed by *Staphylococcus aureus, Serratia marcescens, and Pseudomonas aeruginosa* (two cases each) and*Klebsiella pneumoniae*.

**Table 1:** Procedures performed at each revision (%).

Organism	Revision 1	Revision 2	Revision 3	Revision 4	Revision 5	Revision 6
DAIR/Washout	30 (28)	19 (18)	19 (26)	12 (26)	10 (32)	9 (42)
First stage	66 (62)	45 (42)	19 (26)	18 (37)	6 (19)	6 (29)
Second stage	0 (0)	37 (35)	30 (41)	12 (26)	12 (40)	5 (24)
Single stage	10 (9)	5 (5)	5 (7)	5 (11)	2 (6)	1 (5)
Amputation	0 (0)	0	0	0	1 (3)	
Mean time between revisions in months (SD)	–	10.4 (20.6)	13.0 (18.0)	10.5 (14.0)	8.2 (11.6)	7.6 (10.2)
Total procedures	106	106	73	47	31	21

DAIR stands for debridement and implant retention.

**Table 2:** Number of patients with a change in organism cultured following each revision (%).

	Revision 1	Revision 2	Revision 3	Revision 4	Revision 5	Revision 6
Change in organism	–	46 (43)	43 (59)	27 (58)	12 (39)	13 (62)
Same organism	–	34 (32)	18 (25)	11 (23)	7 (22)	2 (10)
No growth on culture	–	26 (25)	12 (16)	9 (19)	12 (39)	6 (28)
Total	–	106	73	47	31	21

**Table 3:** Number of patients with a change in organism cultured since first revision (%).

	Revision 1	Revision 2	Revision 3	Revision 4	Revision 5	Revision 6
Change in organism	–	46 (43)	53 (73)	37 (79)	26 (84)	19 (90)
Same organism	–	34 (32)	12 (16)	6 (13)	1 (3)	2 (10)
No growth on culture	–	26 (25)	8 (11)	4 (8)	4 (13)	(0)
Total	–	106	73	47	31	21

**Table 4:** Number of patients with polymicrobial infection and multidrug resistance cultured at each revision (%).

	Revision 1	Revision 2	Revision 3	Revision 4	Revision 5	Revision 6
Bacteria (single sp.)	75 (71)	66 (62)	47 (64)	26 (55)	15 (48)	11 (52)
Bacteria (multiple spp.)	22 (21)	12 (11)	8 (11)	10 (21)	4 (13)	3 (14)
Fungus (single sp.)	(0)	(0)	2 (3)	1 (2)	(0)	1 (5)
Bacteria and fungus	(0)	2 (2)	4 (6)	1 (2)	(0)	(0)
No growth	9 (8)	26 (25)	12 (16)	9 (20)	12 (39)	6 (29)
Total	106	106	73	47	31	21
Multidrug resistance (percentages calculated using onlycases with a positive culture)	19 (20)	32 (40)	21 (34)	18 (47)	7 (37)	8 (53)

## 4 Discussion

The principal finding of this study was that patients undergoing multiple revisions for PJI are frequently observed to have a change in organism and sensitivity with each subsequent revision. After six revisions for infection, 90 % of patients have different organisms cultured compared with the initial revision, and 53 % of organisms are multidrug resistant. The proportion of patients with polymicrobial or fungal infection is not associated with number of revision procedures. The proportion of patients with a change in their culture profile between their first and second revision procedure was 43 %, showing the prevalence of a newly cultured organism throughout the series.

A previous study showed that repeat revision for a recurrent PJI had the same organism in only 31.5 % of cases (Zmistowski et al., 2013). The study only characterized a single repeat-revision PJI, rather than our multiply revised patient cohort, but the findings are comparable with our results. After one revision for recurrent PJI, 57 % patients had the same organism cultured; however, after two revisions, 27 % patients had the same organism cultured.

A changing microbiological profile may be due to new intraoperative infection, colonization through a sinus, haematogenous spread, or a false-negative culture from previous surgery either through recent antibiotics or incomplete sampling (Ali et al., 2006). Patients with recurrent PJI frequently have risk factors for infection, such as compromised soft tissues and compromised immune systems. Endoprostheses are often required in multiply revised joints, with a large surface area for colonization and biofilm formation. In fracture-related infection after previous fixation, Corrigan et al. (2022) showed that the timing of the infection relative to the index procedure had no influence on the species of pathogen grown. Certain organisms, particularly fungi, do appear to be more common in multiply revised patients (McCulloch et al., 2023). Within our series there were no fungal infections in all first- or second-revision procedures; however, the incidence was 5 % for the sixth revision, which is much higher than the published incidence of fungal PJI quoted at approximately 1 %–2 % (McCulloch et al., 2023).

Multidrug resistant organisms represented one-fifth of organisms cultured following an initial revision for PJI and over one-half of organisms cultured following six revisions. Therefore, following multiple revision, organisms are more likely to be multidrug resistant. The mechanism of drug resistance is multifactorial, but prolonged antibiotic exposure may lead to selection of microbes resistant to the related antibiotic and bacteria that are hypermutable and, hence, more likely to acquire further antibiotic resistance.

Polymicrobial infection is associated with poorer outcomes from PJI (Kavolus et al., 2019). The proportion of patients with polymicrobial infection did not increase with the number of revisions and represented less than one-quarter of all infections in our cohort. The prevalence of fungal infection was low in this cohort of patients, despite the multiple procedures. Therefore, the number of procedures performed is unlikely to guide the empirical use of antifungals, although none were seen in the first-revision procedure within our series.

The most prevalent organism within our cohort was staphylococcal species, particularly coagulase-negative organisms. Previous studies also found *Staphylococcus* most prevalent, but we did not reproduce the finding of an increased prevalence of atypical infection such as Gram-negative organisms and fungal species following multiple revisions (Benito et al., 2019; Kuiper et al., 2013). Therefore, the number of previous revisions may not be an appropriate guide to select empirical antibiotic or antifungal agents. In addition, one-fifth of revisions in this cohort were for culture-negative infection, and this proportion remained similar even among patients undergoing their sixth revision. Two patients who underwent six revisions had only one organism cultured throughout their surgeries, which was *Staphylococcus epidermidis* in both instances, confirming that it is a challenging organism to eradicate.

When revision surgery fails to eradicate infection, treatment options are observation, antibiotic suppression, or amputation. There was only one amputation in our cohort, consistent with other studies, where amputation for PJI is a rare outcome. In a study of multiply revised total knee replacements, although successful control of infection was only 50 %, there was a limb salvage rate of 97 % during the study period (Rajgor et al., 2022). Our study suggests that antibiotic suppression may be challenging given the prevalence of multidrug resistant organisms following multiple procedures.

Limitations of this study include its retrospective nature and the inherently heterogenous nature of this patient cohort and the surgeries performed. Non-arthroplasty surgery was not recorded, but it frequently proceeds an infected joint replacement with compromise to the soft tissues. We did not explore whether the microbiological profile was associated with patient outcomes. The change in species may be more frequent than our reported 90 % when also considering a likely change in species of coagulase-negative *Staphylococcus*. We defined likely change in species if there were two more changes in antimicrobial sensitivities, as in other studies (Young et al., 2023). Change in the antibiogram may represent acquired resistance through antibiotic exposure, rather than change in species, and sequencing is necessary to address this uncertainty.

## 5 Conclusions

Patients undergoing multiple revisions for PJI are highly likely to experience a change in organism, with 90 % of patients having a different organism cultured by their sixth revision. It is important to administer empirical antibiotics at each subsequent revision, taking into account known drug resistance from previous cultures. Our results do not support the routine use of empirical antifungals.

## Data Availability

A data sharing agreement was made between both clinic departments collaborating on the research project and gained local ethical approval. The approval did not allow for open-access data; therefore. the data itself or the code is not available.

## Appendix A Additional table

**Table A1:** Organisms cultured following each revision.

Organism	Revision 1	Revision 2	Revision 3	Revision 4	Revision 5	Revision 6
No growth	9	26	12	9	12	6
*Staphylococcus aureus*	29	18	7	10	5	4
CoNS	41	40	28	14	7	7
*Streptococcus*	7	2	5	1	1	1
*Enterococcus*	17	6	8	6	6	3
Gram-negative organism	21	24	20	14	6	2
*Cutibacterium acnes*	5	2	0	1	0	1
Other Gram-positive Bacilli	1	2	1	2	0	
*Mycobacterium*	0	0	1	0	0	
Fungus	0	2	6	2	0	1

CoNS stands for coagulase-negative *Staphylococcus*.
